# Dental Service as a Ministry to Life

**Published:** 1900-06

**Authors:** George A. Gordon


### DENTAL SERVICE AS A MINISTRY TO LIFE.[1]

BY REV. DR. GEORGE A. GORDON.[2]

Mr. President and Gentlemen,—I believe that I am responsible for the breadth of this subject, and this was my idea in suggesting it: I heard of a preacher who was in the habit of taking a whole chapter from which to preach his sermon, and when he was questioned why, he said he wanted plenty of room, because if he was persecuted in one text he could flee to another, and I thought there might be some aspect of this subject to which I could flee.

I met a dental epitaph recently which I think will interest you:

> " Here lies the body of Robert Gordon,
> With mouth almighty and teeth accord'n,
> Stranger, walk lightly over this wonder,
> For if he opens his mouth you're gone, by thunder."

Now, this brings to my mind a fact. When Matthew Arnold came to this country to lecture he found that he could not speak. The lamented Professor Churchill, prince among elocutionists, was sent for to see what could be done with him. He said that he found that Mr. Arnold had a very sweet voice naturally, but instead of teeth he had a mouthful of bones. Now, there is one problem suggested by your profession,—What might have been done with Matthew Arnold, and what might be done with others like him?

Several references have been made to the newness of your profession, and the address of President Eliot was upon that theme. It is exceedingly strange that your profession should be new.

---

[1] Read before the union meeting of The New York Institute of Stomatology and the American Academy of Dental Science, Boston, March 21, 1900.

[2] Pastor of the Old South Church.

John Stuart Mill says that theoretic inquiry is stimulated by practical interest. All science has its origin in practical interest; an urgent practical interest is sufficient to organize a new science.

Now I have never had any experience of the pain whatever, but my friends have told me that the toothache is an urgent practical interest, and it is more than strange that this immemorial, pungent interest has been so late in organizing a scientific profession to deal with it. It is a fact, however, and how can we account for it? I think it can be accounted for by another fact, namely, that the pain of toothache has been made light of among almost all peoples. You remember what Robert Burns says in his "Address to the Toothache:"

"When fevers burn, or ague freezes,
Rheumatics gnaw, or cholic squeezes;
Our neighbor's sympathy may ease us
Wi' pitying moan;
But thee—thou hell o' a' diseases,
Aye mocks our groan!"

That statement corresponds to the general experience of mankind. Burns says still more in that remarkable poem. I do not think there is anything like it in all literature. It shows the humanity of the man. He belongs to your brotherhood. He indicates the terrible nature of the pain in several other stanzas. He says:

"Where'er that place be priests ca' hell,
Whence a' the tones o' mis'ry yell,
And ranked plagues their numbers tell,
In dreadfu' raw,
Thou, Toothache, surely bear'st the bell
Amang them a'!"

Then he has an address to his friend the de'il, to whom he talks in a jocose way:

"O thou grim mischief-making chiel,
That gars the notes of discord squeel,
Till daft mankind aft dance a reel
In gore a shoe-thick;—
Gie a' the faes o' Scotland's weal
A towmond's Tooth-ache!"

That is an imprecation equal to any of the Hebrew psalms.

I have indicated one reason for the recency of your profession. People did not care when their neighbors had the toothache; it was only toothache. And then, deeper than that, ignorance. They did not know that pain is a life-killer,—pain of any sort, whether it kills at once or not. Pain disintegrates the organism, it is the enemy of life. And, still further, they did not know, as we all know, that the function of the teeth is most intimately connected with health and life, and it is this immemorial ignorance and want of sympathy that account for the comparative newness of your profession. Men know better now, and they have keener and more humane sympathies, and out of these two facts, stimulated by the great scientific processes of the century bringing exact knowledge to the benefit of life everywhere, has come your profession.

President Eliot suggested to me a remark of Dr. Holmes, that theologians would have modified their views of eschatology if they had constructed their doctrine of future punishment while they were having an attack of toothache. It would be like the story that Tennyson tells of the furnace-tender who went to church and heard a terrific sermon on everlasting torment in hell fire. His wife, a sensitive, conscientious woman, feeling the rebuke of the ideal upon her spirit, feeling, as the best spirits always do, how immeasurably below their ideal they live, almost lost her reason through imagination of the torment which was probably to be hers. The furnace-tender said to her, "My dear, cheer up; 'tain't true, 'tain't true; no constitution could stand it." And it is well to take all doctrines of speculation into the vivid, instructed humane consciousness of man, and make them tell their story there, and receive such revision as will send them out upon a new mission.

President Eliot was perfectly right in speaking of the entire newness which has come over the world when he alluded to the churches. Everything is new. The fact is, wherever you find a stationary interest, there you find a decaying interest. Wherever the interest is being pushed into commanding power and influence, there is novelty, there is renewal; and wherever this note is absent, there is retrogression.

I looked at the titles of these two societies,—the mouth, teeth, stomach, and the soul,—and I thought that the dreadful thing would be not to have a congregation with teeth, but to have one without teeth. What could you do with such a congregation? Bad digestion, eternal dyspepsia; could it issue in anything other than unhappy, pessimistic souls, impatient, impossible? President Eliot never could get a dollar for the support of the University out of such a constituency as that. Teeth are tremendous; the want of teeth still more tremendous.

I had expected that much would be said about the influence of your profession in making human life attractive. Your profession is a fine art. The fact is, everything is beautiful that is wholesome, clean, healthy, entire, and the finest features in the world are absolutely spoiled by a toothless mouth; and as a great deal of good fellowship depends upon the attractiveness of human beings to human beings, I look for a ministry to life from your profession, not simply in the cases of disease and loss, but still more in taking the children in hand and making their teeth strong and lasting as existence.

I was born in Scotland, and notwithstanding the fact that Burns wrote a poem on the toothache, there was very little toothache there then; there was very little of it until the new diet was introduced to which President Eliot referred. Toothache was never heard of in the family in which I was born. I do not think I had a dentist look into my mouth until I was thirty-five years old. No need of it. Now, if the dental profession could abolish itself in this way, what a tremendous service it would render. That is what we are all aiming at. The time is coming when there will be no need of my profession. Holiness written on the harnesses of the horses, a city without a church. Boston is, however, still a good way off from that. And a good way off from the necessary abolition of your profession.

Seriously, we speak of the coming man and the coming woman, and we speak of the coming human being, and he is our supreme interest, and every serious worker in every high profession must occasionally lift up his eyes unto that hill whence cometh his inspiration. It is a great inspiration to think that by righteous living, by honorable and skilful service and beneficent work, something may be done to improve the quality of the human race in every respect, and it seems to me that this your profession, through generations of service, enlarging science, and growing skill, may contribute to that coming man; not in one aspect of his life alone, but through health and integrity and attractiveness and happiness, to the very highest element in manhood, character, and spirit.

